# Ablation of Myocardial Tissue With Nanosecond Pulsed Electric Fields

**DOI:** 10.1371/journal.pone.0144833

**Published:** 2015-12-14

**Authors:** Fei Xie, Frency Varghese, Andrei G. Pakhomov, Iurii Semenov, Shu Xiao, Jonathan Philpott, Christian Zemlin

**Affiliations:** 1 Department of Electrical and Computer Engineering, Old Dominion University, Norfolk, Virginia, United States of America; 2 Center for Bioelectrics, Old Dominion University, Norfolk, Virginia, United States of America; 3 Department of Surgery, Eastern Virginia Medical School, Norfolk, Virginia, United States of America; Rutgers University -New Jersey Medical School, UNITED STATES

## Abstract

**Background:**

Ablation of cardiac tissue is an essential tool for the treatment of arrhythmias, particularly of atrial fibrillation, atrial flutter, and ventricular tachycardia. Current ablation technologies suffer from substantial recurrence rates, thermal side effects, and long procedure times. We demonstrate that ablation with nanosecond pulsed electric fields (nsPEFs) can potentially overcome these limitations.

**Methods:**

We used optical mapping to monitor electrical activity in Langendorff-perfused New Zealand rabbit hearts (n = 12). We repeatedly inserted two shock electrodes, spaced 2–4 mm apart, into the ventricles (through the entire wall) and applied nanosecond pulsed electric fields (nsPEF) (5–20 kV/cm, 350 ns duration, at varying pulse numbers and frequencies) to create linear lesions of 12–18 mm length. Hearts were stained either with tetrazolium chloride (TTC) or propidium iodide (PI) to determine the extent of ablation. Some stained lesions were sectioned to obtain the three-dimensional geometry of the ablated volume.

**Results:**

In all animals (12/12), we were able to create nonconducting lesions with less than 2 seconds of nsPEF application per site and minimal heating (< 0.2°C) of the tissue. The geometry of the ablated volume was smoother and more uniform throughout the wall than typical for RF ablation. The width of the lesions could be controlled up to 6 mm via the electrode spacing and the shock parameters.

**Conclusions:**

Ablation with nsPEFs is a promising alternative to radiofrequency (RF) ablation of AF. It may dramatically reduce procedure times and produce more consistent lesion thickness than RF ablation.

## Introduction

Ablation of myocardial tissue is an essential technique for the treatment of cardiac arrhythmias[1,2]. It can be used to directly ablate arrhythmogenic tissue or atrial locations that exhibit complex fractionated atrial electrograms [3]. Ablation can also be used to create nonconducting lesions, which can be placed around arrhythmogenic tissue to contain the arrhythmic activity, or between anatomical landmarks to interrupt reentrant pathways [4]. The most common use for cardiac ablation is in the treatment of atrial fibrillation (AF) and flutter, but it is also important in the ablation of ventricular tachycardia [5].

Myocardial tissue is most commonly ablated by application of radiofrequency currents via monopolar endocardial catheters. The RF currents lead to resistive heating, especially in the vicinity of the catheter electrode, and eventually to necrosis of tissue around the catheter due to hyperthermia. The duration of RF application is calibrated to obtain ablation all the way through the cardiac wall. By placing individual RF ablations side by side, non-conducting lesions are created. RF ablation achieves excellent acute success rates, e.g. the acute isolation of the pulmonary veins is almost always achieved [6].

The most important limitation of RF ablation is its high recurrence rate, especially for non-paroxysmal AF [7,8]. AF recurs within months to years after the ablation, and recurrence is commonly attributed to gaps in the lesions [9]. Control over the lesion depth and geometry is limited, because RF energy is applied from the endocardial surface, so the lesion profile is inherently variable from endo- to epicardium, and even irrigated catheter tips cannot completely solve this problem [10]. Furthermore, blood vessels act as a heterogeneous cooling network during RF ablations and further limit the consistency that is achievable [11].

RF ablation has a significant complication rate (approximately 4%), and complications include stroke, tamponade, vascular injury, pulmonary vein occlusion, and atrioesophageal fistulae [12–14]. Many of the complications may be related to the generation of heat during RF application. Also, RF procedures are lengthy, on the order of 2–3.5 hours [15,16], which poses a general surgical risk and ties up hospital resources thus limiting patient throughput and EP lab efficiency.

A more recent alternative to RF ablation is cryoablation. Cryoablation is faster [17], and thermal complications associated with RF may occur at lower rates [18], while the effectiveness is comparable to that of RF ablation [19].

Here, we demonstrate an alternative physical ablation mechanism that is based on applying very short (nanosecond) pulsed electric fields (nsPEFs) and may overcome the limitations of RF- and cryoablation. Ablation with nsPEFs kills the targeted cells by porating their membrane, allowing calcium and other components of the extracellular medium to enter the cell. It is non-thermal, fast, and gives better control over the lesion geometry than thermal ablation. Ablation with nsPEFs has been successfully used for tumors [20], but not yet for cardiac tissue.

The use of nanosecond pulses is motivated by their distinct mechanism of interaction with the cell membrane potential, with is markedly different from that of longer (millisecond or above) pulses. In conventional stimulation, membranes act as barriers for the movement of free electrolyte charges in the externally applied electric field. The capacitive charging of membranes amplifies the external field by a factor of several thousands (known as the membrane amplification factor) [21–23]. This amplification enables the electroporation of cells by external fields which are many orders of magnitude weaker than the local transmembrane field required for electroporation. In a syncytial tissue, the electrical connections between cells restrict the amplification; hence it is predominantly the areas at the cathode and at virtual cathodes formed by electrical inhomogeneities that experience electroporation when the E-field is turned on [24].

In contrast, nsPEF stimuli are too brief for capacitive charging, and displacement currents dominate over conduction currents [21,25]. Therefore intercellular electric connections do not affect (or just minimally affect) membrane charging, and every cell even in the syncytial tissue behaves as an independent entity. This situation is electrically equivalent to each cell having its own virtual cathode, and electroporation is not affected by the vicinity to the cathode. Instead, all cells within a high enough electric field area. For the same reason (not relying on conduction currents), nsPEF penetrates deeper and the E-field distribution in tissue is less affected by electrical inhomogeneities [21,25,26], resulting in more uniform ablation.

In this study, nsPEFs are applied via two thin parallel wire electrodes that penetrate the myocardial wall and ablate the volume between the electrodes. Lesions are created by repeated application (as in the case of RF ablation).

## Methods

### Surgical preparation

The IACUC of Old Dominion University reviewed and approved our animal protocol for the experiments on which we report (protocol number 13–017). New Zealand white rabbits of either sex (3–4 kg, n = 12) were heparinized (500 IU/kg) and brought to a surgical plane of anesthesia with 2.5–4% isoflurane. The heart was rapidly removed, the aorta cannulated and flushed with ice cold Tyrode solution (in mM: NaCl: 128.2, NaCO_3_: 20, NaH_2_PO_4_: 1.2, MgCl_2_: 1.1, KCl: 4.7, CaCl_2_: 1.3, glucose: 11.1), and the heart was placed in a Langendorff-perfusion setup, where it was perfused and superfused with warm oxygenated Tyrode solution (37±0.5°C) at a constant pressure of 60–80 mmHg. After 30 min equilibration, 10–15 mM of 2, 3-butanedione monoxime was added to eliminate contractions.

### Optical mapping

The preparation was stained with the near-infrared dye DI-4-ANBDQBS. A stock solution was made by dissolving 10 mg dye in 1.2 ml of pure ethanol; for each experiment, 30 μL of the stock solution was diluted with 15 mL of Tyrode's solution and injected as a bolus. A 1000 mW, 671 nm diode laser (Shanghai Dream Lasers) was directed through a 5 degree conical diffusor and then through a dichroic mirror (λ_crit_ = 690 nm) onto the heart to achieve uniform illumination. Fluorescence light passed the dichroic mirror and a 715 nm long pass filter and was recorded with a CCD camera (“Little Joe”, SciMeasure, Decatur, GA) at 1000 frames per second.

### Ablation electrodes and choice of ablation sites

**Fig 1:**
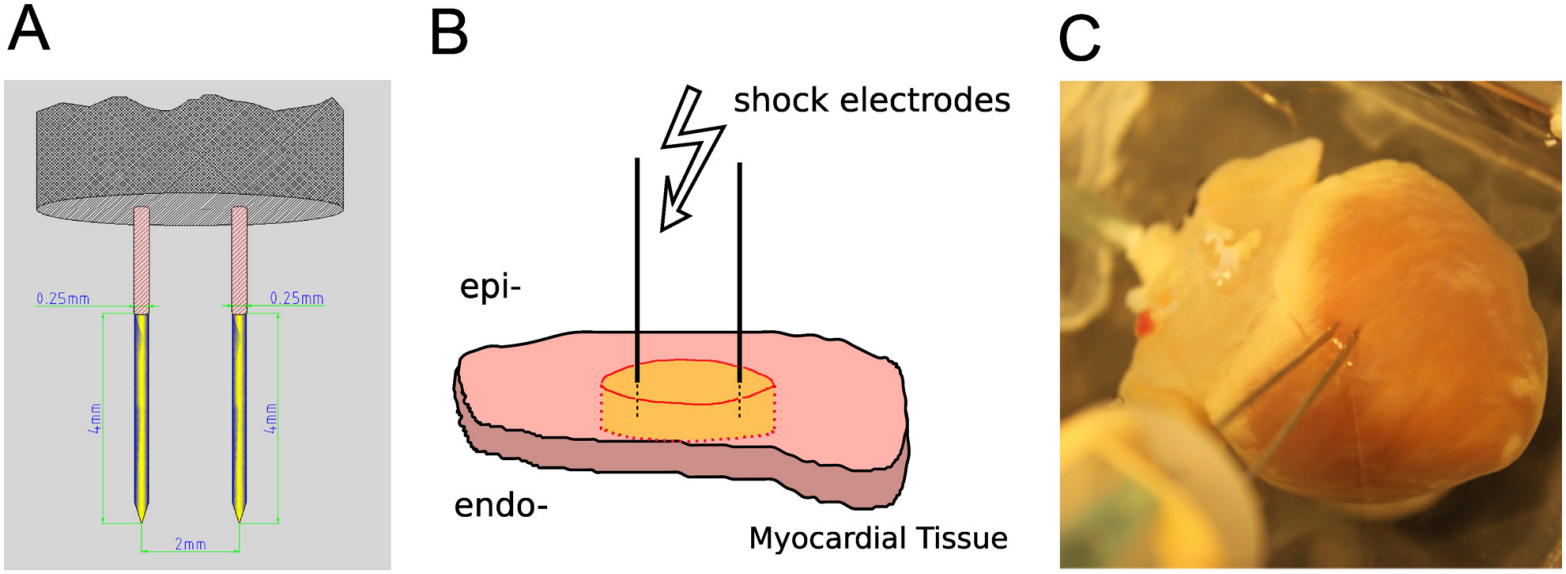
Electrode configuration for nsPEF ablation. **A**: Electrode geometry: Two parallel 250 μm tungsten needles uninsulated at the tip (yellow segment). **B**: Electrode placement in the tissue. A slab of cardiac tissue (pink) is penetrated by two needle electrodes. Upon shock delivery, an area between and around the electrodes is ablated (yellow). **C**: Electrode placement in a rabbit heart. Electrodes were inserted from the epicardium, penetrating all the way through the myocardial wall.

Ablation electrodes were made of two parallel 250 μm tungsten needles (see Fig 1A). The electrode spacing was adjustable from 2 to 6 mm, and the electrodes were uninsulated over the terminal 4 mm and sharpened at the tip. Electrodes were dipped into surgical ink to mark the insertion sites and then inserted into the right or left epicardium (see Fig 1B), so that they penetrated the entire ventricular wall. Ablation sites were chosen at least 1 cm away from the septum. When linear lesions were desired, 4–5 consecutive ablations were performed next to each other to create a linear lesion of 12–18 mm length.

### Pulse generation

**Fig 2:**
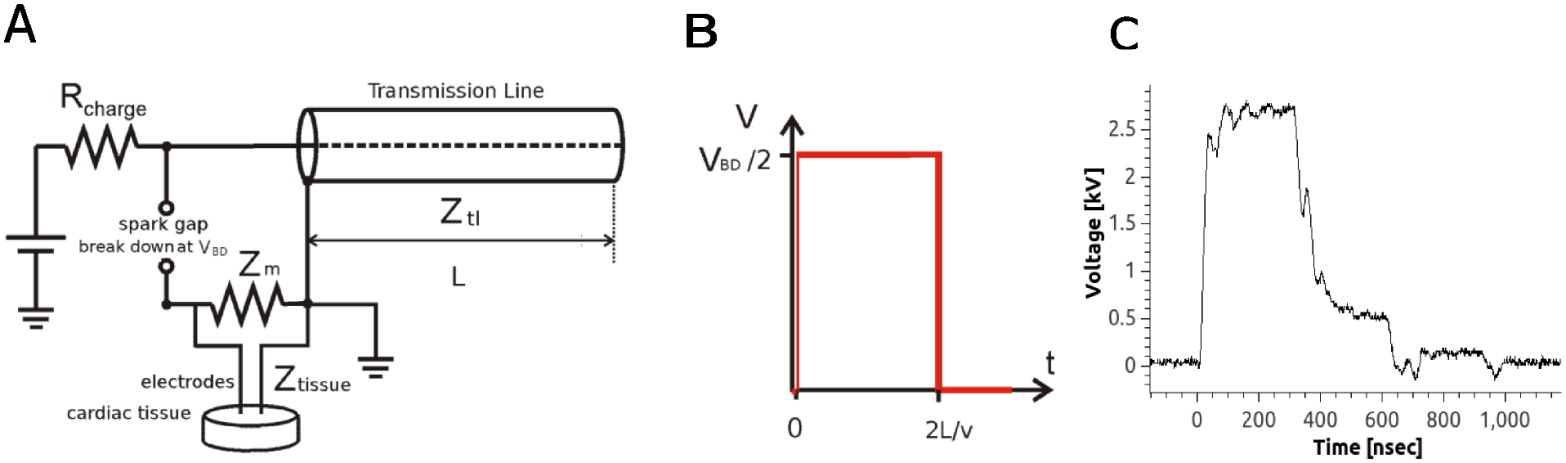
Electric pulse generation and shape. **A**: Pulse generation with a transmission line generator. R_charge_ is the charging resistor, Z_tl_ is the impedance of the transmission line, Z_tissue_ is the impedance of the tissue, and Z_m_ is the additional impedance added in parallel to the tissue in order to match the impedance of the load to that of the transmission line. V_BD_ is the breakdown voltage of the spark gap, adjustable by changing its width and L is the length of the transmission line. **B**: Theoretical pulse shape, for the diagram in Panel A, where v is the speed of light in the transmission line. Pulse duration is proportional to the length of the transmission line in Panel A. **C**: Experimentally measured pulse shape.

Nanosecond pulses were created with a transmission line generator (see Fig 2A). A double shielded coaxial cable (RG-217U) was used as a capacitor (C = 3.1 nF). An additional resistor *Z*_*m*_ = 13.7Ω was placed in parallel with the heart to achieve impedance matching between the transmission line and the load. In theory, this setup should charge the transmission line until the breakdown voltage of the spark gap is reached and apply rectangular pulses of duration t = 2l/v to the load, where l is the length of the transmission line and v the speed of light in the transmission line. In our case, l = 35 m, v = 0.66 c (c is the speed of light), and consequently, t≈350 ns [27] (see Fig 2B). The actual pulse shape was recorded with an oscilloscope (Tektronix 1001B, Beaverton, OR) and followed the theoretical prediction with good accuracy (see Fig 2C).

### Ablation protocol

At each ablation site, we applied trains of 350 ns pulses (either 6 pulses at 3 Hz of 20 pulses at 1 Hz) of different amplitudes. We used an adjustable spark gap to allow the adjustment of the pulse amplitude, the pulse frequency was adjusted by adjusting the supply voltage, provided by a 0–20 kV power supply (EH Series, Glassman, Highbridge, NJ). When we generated trains of pulses, the pulse amplitude was reproducible within ± 0.2 kV over the amplitude range used in this study.

### Lesion analysis

For each element of the lesion, the two surface insertion points A and B of the electrodes were identified. At the midpoint of the line AB, the extension of the lesion perpendicular to the line AB was considered the width of the lesion segment. The width of the lesion was defined as the average of the widths of the segments.

### PI/TTC staining and sectioning

After the creation and electrophysiological evaluation, preparations were stained either with propidium iodide (PI, 30 mM/30 min) or tetrazolium chloride (TTC, 30 mM/20 min), for further study of the geometry of the ablated volume. For PI stains, we subsequently washed out the PI using our coronary perfusion for 40 minutes, leaving only the cells with compromised membrane stained with PI [28].

For both TTC and PI stains we cut lesions out of the tissue preparation and mounted each lesion separately in a block of agar. We then sectioned the lesions (section thickness: 300 μm). For PI stains, we recorded the fluorescence (illuminated at λ = 532 nm) for each section. By defining a fluorescence threshold that corresponds to dead tissue, the ablated area was identified.

### Modeling of field distribution

We used the COMSOL Multiphysics package (COMSOL, Palo Alto, California) for finite element modeling of field distributions. We modeled the tissue as a circular, homogeneous sheet with uniform conductance and a radius of 4 cm. At the electrode locations, the sheet had two circular holes of 250 μm diameter (the electrode diameter), spaced 2 mm apart. The Laplace equation, Δ*V* = 0, was solved on the tissue domain for the transmembrane voltage. The boundary conditions were that at the interior (electrode) holes, *V* equaled the respective applied electrode potentials, and at the outer boundary, *V* was zero.

## Results

### Single nsPEF ablations create conduction block

**Fig 3:**
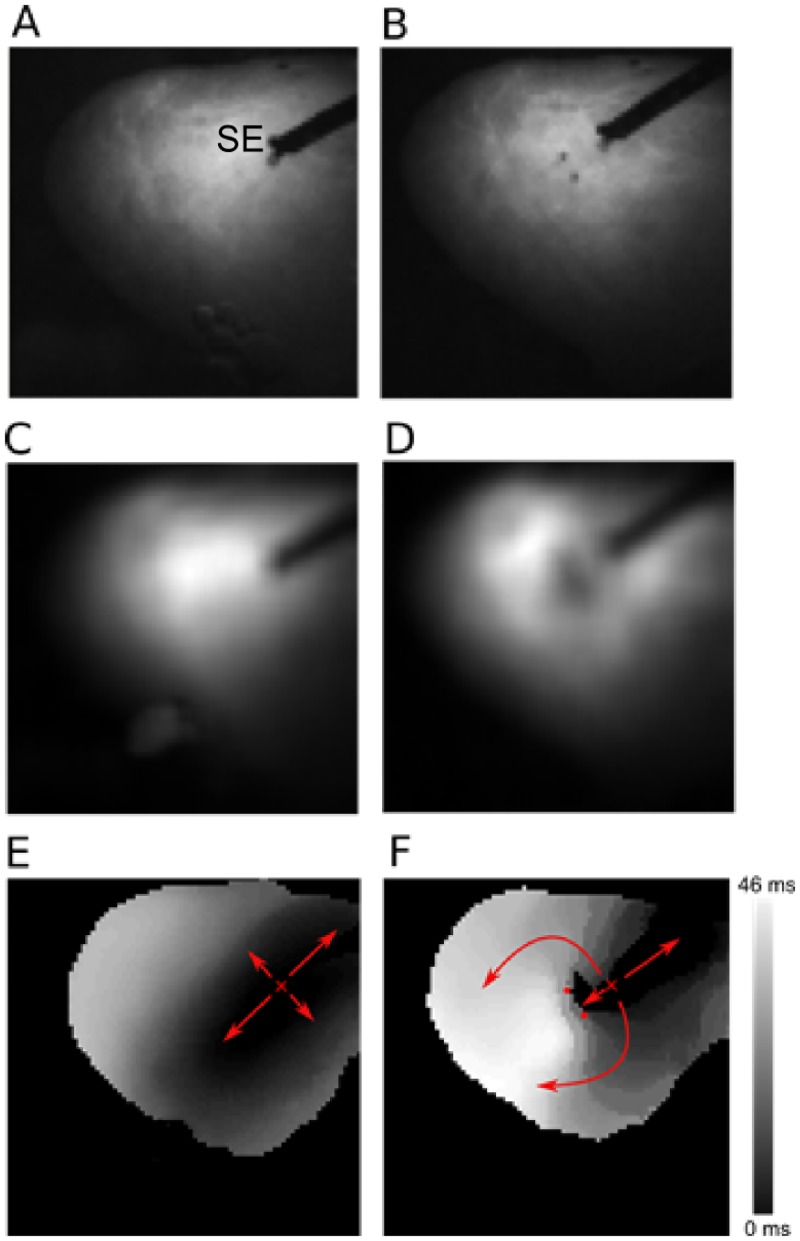
Propagation of excitation before and after nsPEF application. **A:** Photograph of the cardiac surface before ablation. The stimulation electrode (marked “SE”) is used to initiate electrical activation. **B:** Photograph of the cardiac surface after ablation. The electrode insertion points are marked by black dots. **C:** Action potential amplitude map before shock application. Black corresponds to zero action potential amplitude, white to maximal action potential amplitude. **D:** Action potential amplitude map after shock application. **E:** Activation map before shock application. Colors code the time after stimulus application at which a surface element is activated. Black areas are activated first, white areas last (see scale). Small red “x” marks the stimulation site, arrow indicate the direction of propagation. **F:** Activation map after shock application. Activation is blocked at the site of shock application (small red dots indicate shock electrode positions.

Ablation with nsPEFs consistently created conduction block if the field strength was sufficient. Fig 3 shows a representative example of conduction block creation, recorded with optical mapping. Panel A shows a photograph of the cardiac surface, including the stimulation electrode. Panel B shows the same cardiac surface after nsPEF application, with two spots of surgical ink indicating the positions of the ablation electrodes. Panels C and D compare the action potential amplitudes (obtained via optical mapping) before and after nsPEF application, showing that the action potential amplitude goes to zero in the region surrounding the nsPEF electrodes. Panels E and F illustrate the propagation of excitation before and after nsPEF application. Before the application, excitation propagates in all directions, with elliptical isochrones whose long axis corresponds to the local fiber direction. After the shock, propagation in the direction of the nsPEF application is blocked, so that excitation propagates around this electrically inactive region, as shown by the smoothly changing activation times along this path, to eventually excite the tissue on the other side of the nsPEF ablation. Note that the data in Panel F do not exclude the possibility that the observed block is really just very slow conduction (which would excite the tissue on the other side of the lesion after the wave that went around the lesion arrived). This interpretation appears, however, contrived in light of the absence of electrical activity in the treated region (Panel D) and the evidence of cell death in this region presented below.

**Fig 4:**
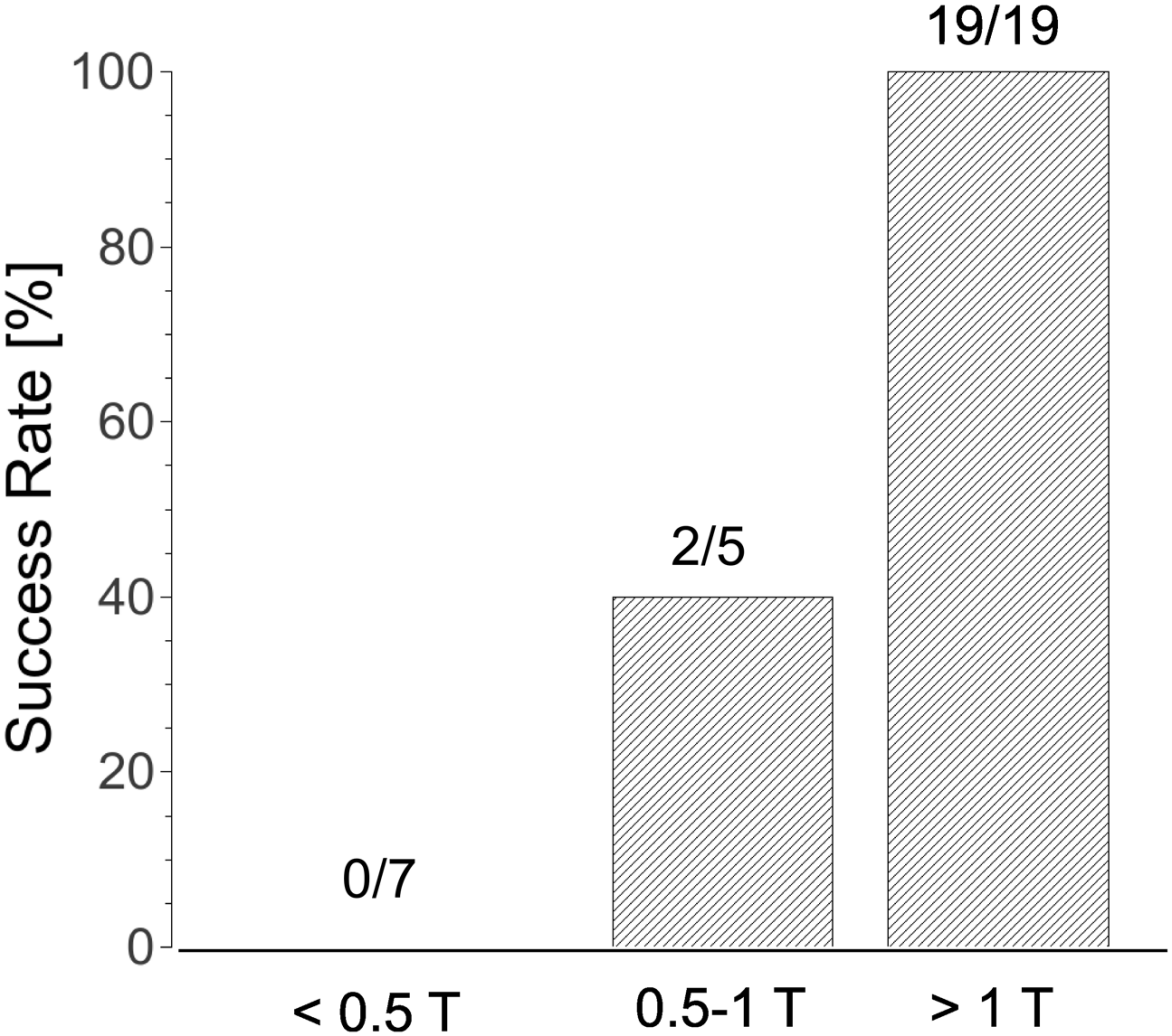
Success Rate of nsPEF ablation for different field strengths. The threshold field strength (T) was 2.3 kV for 2.3 mm electrode separation and 4 kV for 4 mm electrode separation. The bars show combined results for both field strengths, relative to the thresholds.

The field necessary for ablation depends on the shock parameters and electrode spacing (see Fig 4). For our default parameters (2.3 mm spacing, 6 shocks at 3 Hz), field strengths above 2.3 kV consistently caused block (n = 19). When we changed to 20 shocks at 1 Hz, a lower pulse amplitude of 2.0 kV was consistently sufficient (n = 5). For an electrode separation of 4 mm (20 pulses at 1 Hz), a pulse amplitude of 4 kV was consistently sufficient (n = 3).

In general, we found that pulse amplitudes above half the thresholds mentioned above were still sometimes successful in creating block (2/5), while shock amplitudes below 50% of the thresholds stated above never succeeded in creating a lesion (n = 7). All these data are from single ablations; they are complemented by the data for longer lesions below (a blocking lesion implies that all individual ablations are blocking).

### Effect of shock amplitude on geometry of ablated volume

**Fig 5:**
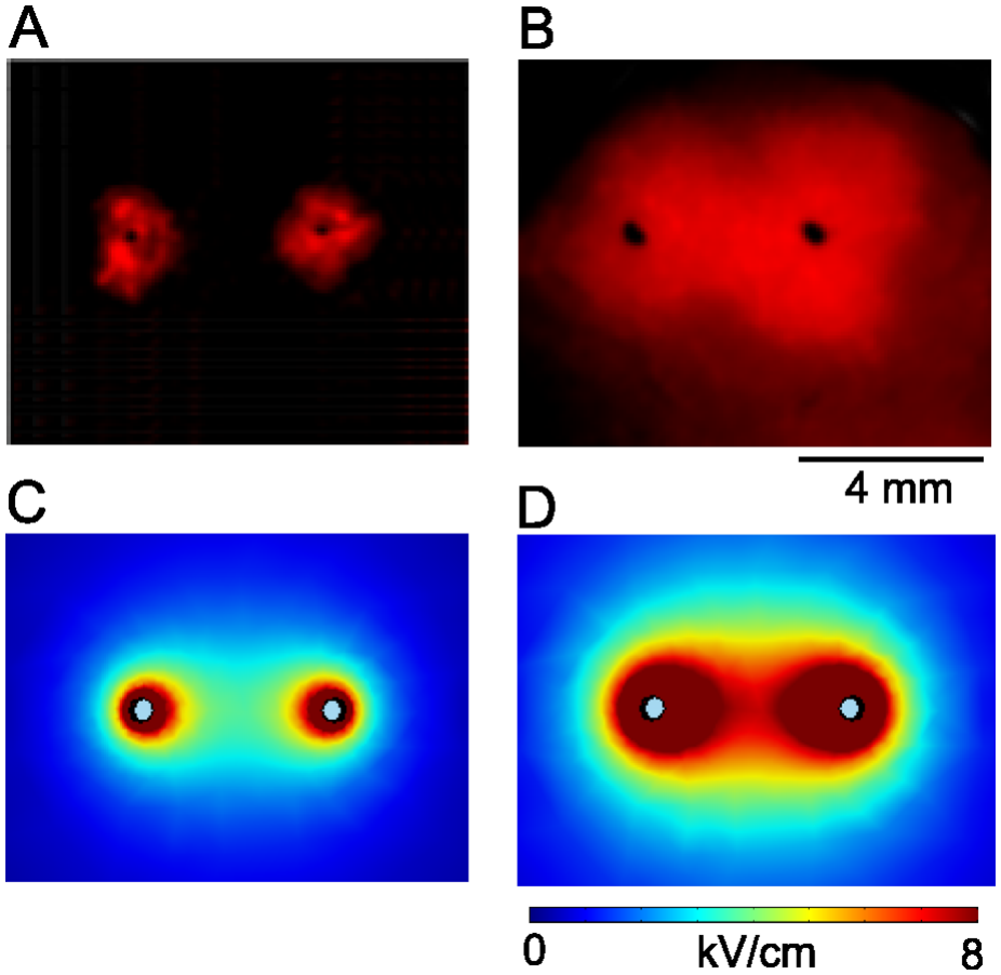
Ablated volume varies with shock amplitude. Black dots indicate the positions of the ablation electrodes, surface fluorescence of propidium iodide (red) shows which part of the tissue has been ablated. Ablation electrodes are 4 mm apart. **A:** Shock amplitude 1 kV, **B:** Shock amplitude 2 kV. C: Computed field distribution (|E|) for a 1 kV shock. D: Computed field distribution for a 2 kV shock.

When we varied the shock amplitude while keeping all other shock parameters fixed (4 mm electrodes spacing, 20 pulses at 1 Hz) and subsequently stained the tissue with PI, we found a characteristic dependency of the geometry of the ablated volume on shock amplitude, shown in Fig 4. For a small shock amplitude (1 kV/cm), we observe ablation only in limited areas around the ablation electrodes (see Fig 5A). For a larger shock amplitude (2 kV/cm), the areas around the electrodes merge so that a contiguous lesion is created (see Fig 5B).

Computed field distributions for both 1 and 2 kV/cm are shown in Fig 5C and 5D. They support the idea that tissue dies whenever the local field strength is above a critical threshold. Comparing Fig 5A and 5C and Fig 5B and 5D, we estimate that this critical threshold should be in the range of 3–5 kV/cm.

### Longer lesions with multiple nsPEF applications

**Fig 6:**
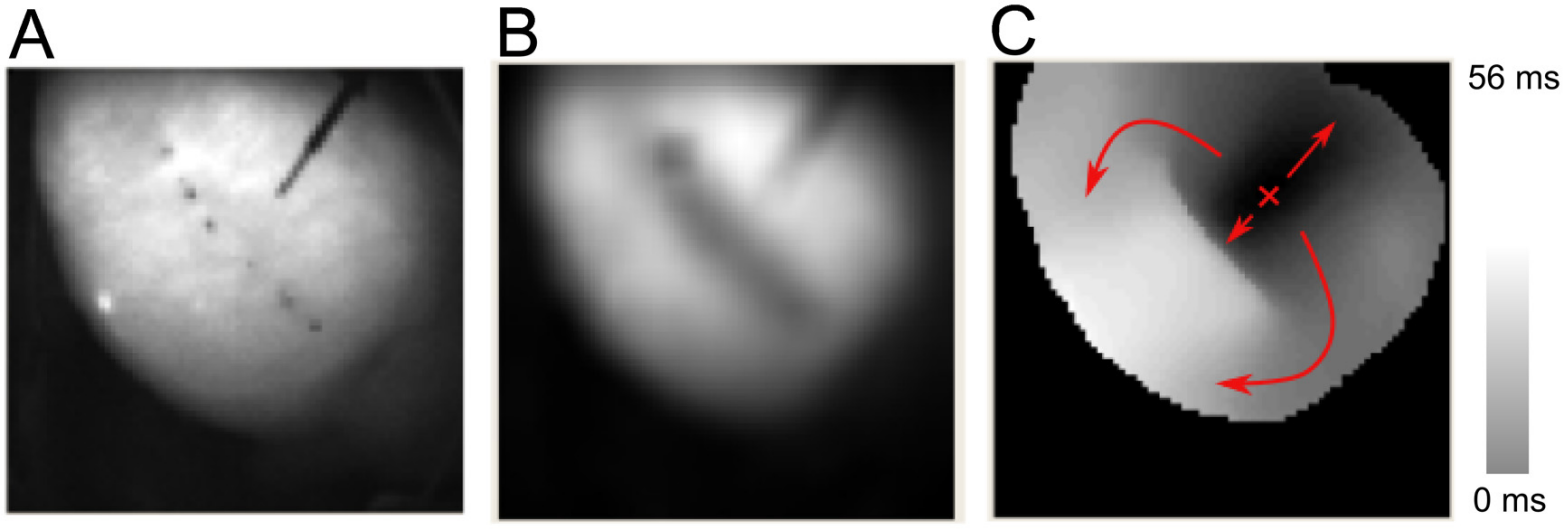
Evaluation of nsPEF lesion (compare Fig 3 for details). **A:** Photograph of the cardiac surface after ablation. The pairs of black dots mark the locations of the (successive) positions of the ablation electrodes, the black diagonal line in the upper right is the stimulation electrode. **B:** Action potential amplitude map after ablation. **C:** Activation map after ablation.

We created longer lesions by placing single nsPEF ablations along a line, just like lesions are created using RF ablation. For electrode spacing 2.3 mm, our lesions consisted of 4–5 single ablations. The subsequent electrode positions were shifted ~ 0.7 more by ~3 mm, so that the gap was ~0.7 mm and the lesion length was ~15 mm. For electrode spacing 4 mm, we used 3–4 single ablations and electrode positions were shifted by 4.7 mm, leading to lesion lengths of ~16 mm. Based on our experience with single application, we chose set our standard protocol to 6 pulses at 3 Hz. The field strength was set to 2.3 kV for 2.3 mm electrode separation and to 4.3 kV for 4 mm electrode separation (i.e. ~10 kV/cm if calculated simply as "voltage over distance"). A representative example with 2.3 mm electrode separation is shown in Fig 6.

Panel B shows that electrical activity has ceased where nsPEF was applied. Panel C shows that activation propagates around the treated region (as indicated by the arrows), and the smooth change in activation time indicates that this propagation happens with approximately constant speed. While activation also propagates from the stimulation site towards the lesion, the abrupt jump in activation time at the lesion is strong evidence that conduction block has indeed been achieved.

### Lesion statistics

**Fig 7:**
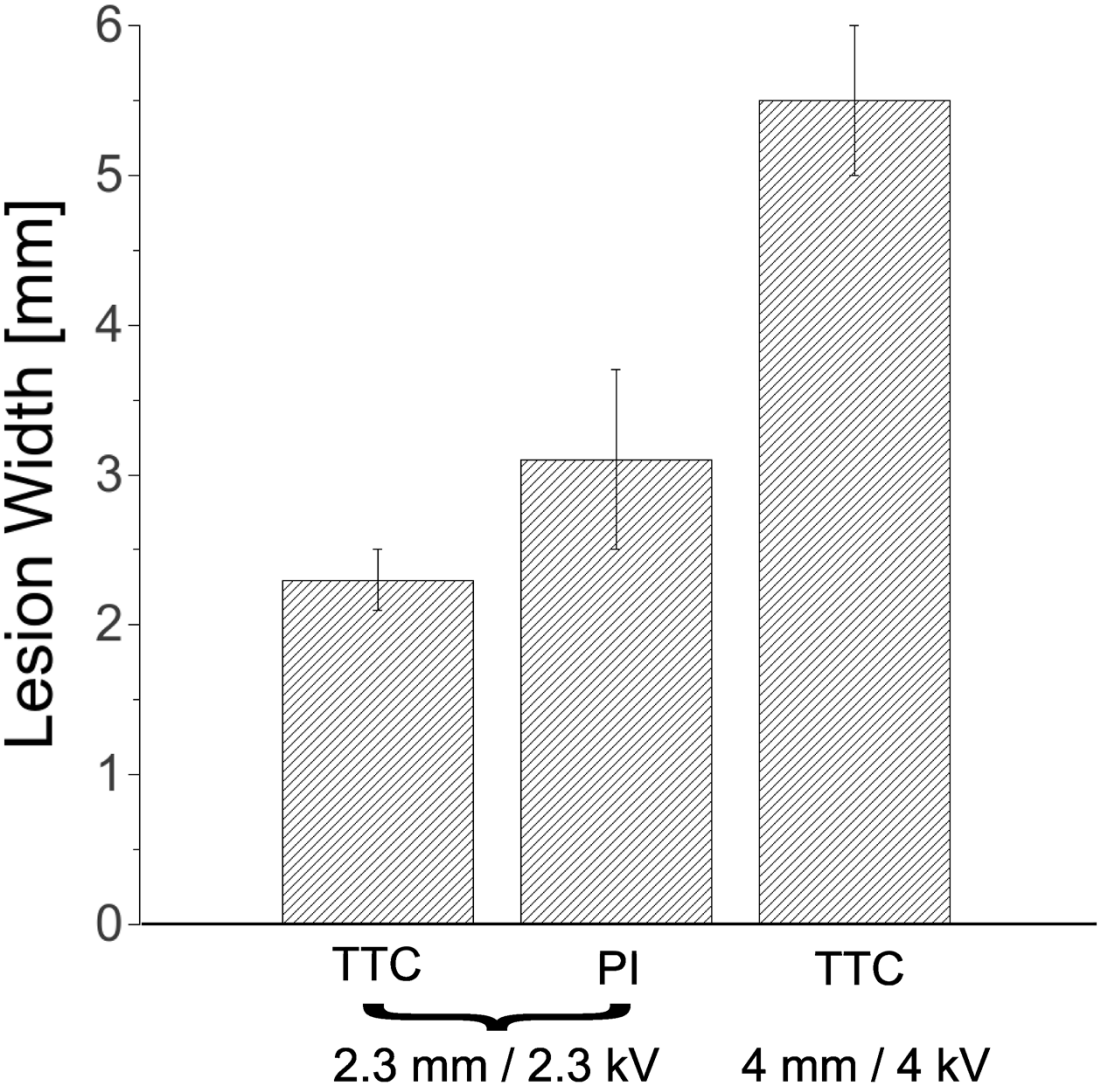
Lesion width statistics. For shocks of 2.3 kV over 2.3 mm, we evaluated lesion width in some hearts with TTC staining and in other hearts with PI staining. For shock of 4 kV over 4 mm, we evaluated lesion width in all hearts with TTC staining. Bar heights show averages, error bars indicate standard deviations.

We created a total of 16 lesions in 12 rabbit hearts. Based on our single ablations, we expected consistent ablation success with low individual ablation times (< 2 s), and indeed, all lesions created were nonconducting (16/16). For 2.3 mm electrode separation, we assessed the lesion width both with PI stains and TTC stains (see Fig 7). For PI stains (n = 7) we found an average width of 2.3+/-0.2 mm, while with TTC stains (n = 7), the average was 3.1+/-0.56 mm. For 4 mm electrode separation (n = 2), the evaluation of the lesion with TTC staining gave a thickness between 5 and 6 mm.

### 3D Geometry of lesions

**Fig 8:**
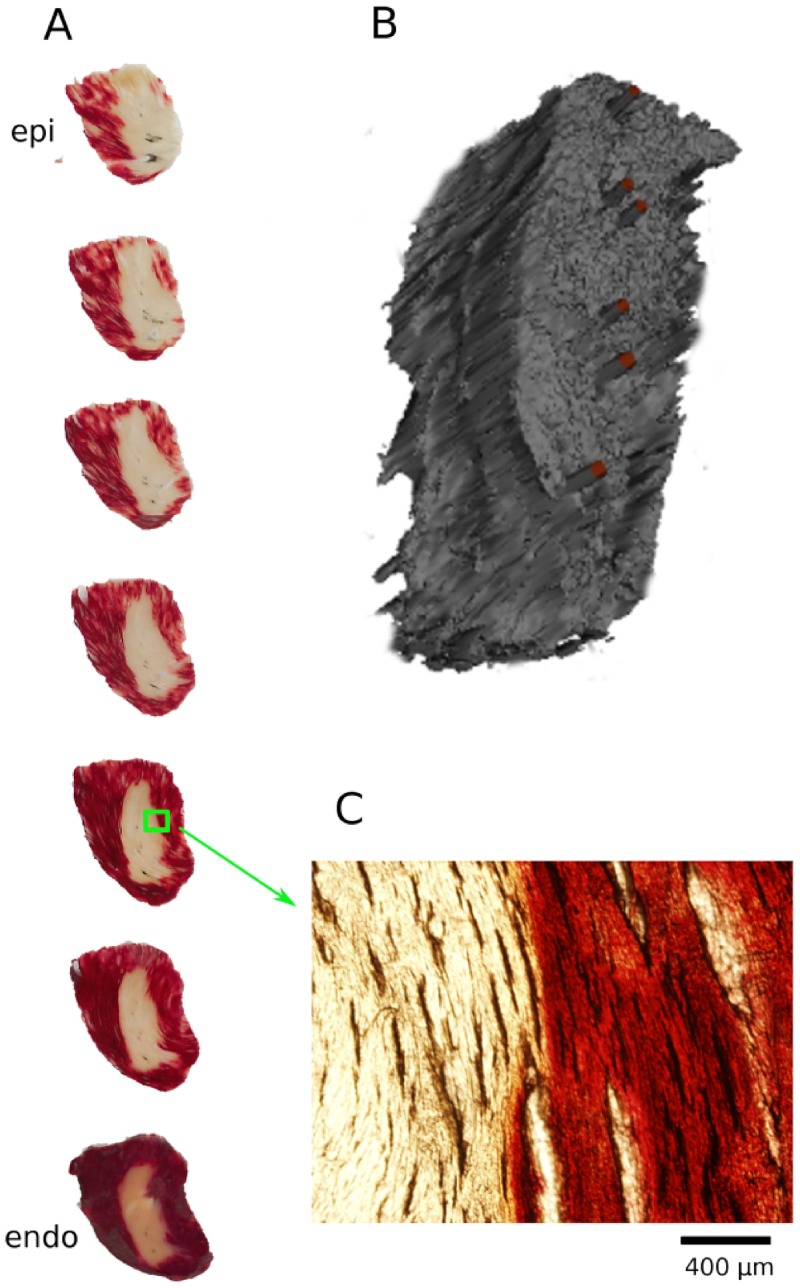
3D reconstruction of the geometry of the ablated volume. **A:** A stack of TTC-stained sections of the lesion. White and red areas identify dead and live tissues, respectively. Sections are 300 μm thick. **B:** Three-dimensional rendering of the lesion geometry, obtained from the sections in Panel A. The red bars indicate the successive positions of the ablation electrodes. **C:** Zoom into one of the TTC-stained sections at the boundary of the ablated volume.

We also investigated the 3D geometry of selected lesions we created. Fig 8 shows TTC staining in a series of sections from epi- to endocardium (Panel A) and a 3D reconstruction of the lesion geometry (Panel B). The section geometry is far more consistent from epi- to endocardium than is typical for RF or cryoablation [10]. Also, the boundary of the ablated region is very sharp; the zoomed picture (Panel C) shows that even at the level of individual cardiac fibers there is a very abrupt transition from unstained to almost fully stained tissue.

### Ablation Speed

We were able to create non-conducting lesion with 6 pulses delivered at 3 Hz in every attempt. This corresponds to a treatment time of 1.67 s per location. We also tried to achieve ablation with single pulses, but even at field strengths of 5–6 kV, the maximum that our generator could supply, we were not able to create lesions consistently.

### Thermal effects

The amount energy deposited per pulse can be computed from the shock parameters and the generator capacitance (3.1 nF), and it is less than 8 mJ (for 2.3 kV, a typical pulse amplitude). Even with 20 pulses the total energy is below 200 mJ (compared to typically ~1,000 J per site for RF ablation). In direct experimental measurements at the midpoint between the electrodes, we were not able to detect any temperature increase after the shock, indicating that the increase was smaller than ~0.1°C.

## Discussion

We have demonstrated that using nsPEFs, we can reliably ablate myocardial tissue with more consistent lesion cross section than RF/cryoablation, in a fraction of the time, and without thermal side effects. Lesion width was consistent and could be adjusted between 2 and 5.5 mm by choosing different electrode distances and pulse amplitudes.

### Implications for recurrence

Atrial fibrillation usually recurs when pulmonary vein (PV) isolation lesions become conductive again [9]. While the precise mechanism for the loss of lesion integrity is not known, it is reasonable to assume that lesions most easily become conductive at locations at which they are particularly narrow (or even have a gap). It appears that a promising approach to prolong lesion integrity is to make lesions more uniform in width, specifically avoiding that the width is below some minimum width anywhere along the lesion. Our data suggest that nsPEF ablation may give electrophysiologists the tools to achieve such greater lesion uniformity.

### Absence of thermal effects

The complete absence of thermal effects and the associated complications side effects is a major advantage of nsPEF ablation over RF and cryoablation. RF ablation suffers from significant rates of major complications [12,13]. Esophageal injuries are common [29] and can in extreme, rare [30] cases lead to fistulae, which are associated with high mortality [31]. PV stenosis is another important complication that was reported to occur in 0 to 19% (mean, 2%; median, 3.1%) after 2004 [32]. It is possible that the nsPEF rates for PV stenosis will be lower (PV stenosis is a consequence of the inflammatory response [33,34], which may be aggravated by heating). Compared to cryoablation, the elimination of phrenic nerve palsy [35] would be a major improvement. For stroke, another important complication of thermal ablation, it is less clear how often it is a direct consequence of thermal effects, but a since nsPEF ablation excludes the risks of charring and thrombus formation, it should provide some benefit here as well.

### Speed of applications

Since nsPEF ablation requires only requires less than 2 seconds per ablation site, it is realistic to assume that overall procedure times, e.g. for AF ablation, can be reduced substantially. Such a reduction will reduce the overall surgical risk, reduce the stress on patients, and free up resources of the hospitals that are expected to perform more and more ablation procedures.

### Effect of shock amplitude on lesion geometry

Our experimental finding that smaller shock amplitudes lead to ablation only in the vicinity of the electrodes, while larger shock amplitudes ablate the whole region between the electrode is consistent with similar experiments performed in 3D in-vitro tumor models [36] as well as computations of field distributions [37]. For 3D in-vitro tumor models, a critical field that predicted cell death was also identified [36].

While we get good qualitative agreement between our experiments and model, we recognize that our model excludes important electrical properties of the myocardium, in particular its anisotropic conductivity and the fact that the direction of highest conductivity changes across the myocardial wall (”twisted anisotropy”). We are in the process of developing a model that includes these features of cardiac tissue.

### Comparison to DC and pulsed electric fields of longer duration

There is a long history of DC ablation, in fact DC used to be the standard modality of thermal ablation before it was established that RF has lower complication rates [38,39]. Also, pulsed electric fields have been used before for ablation of cardiac tissue [40–42], although these studies used longer pulses (in the micro- and millisecond range) and much higher ablation energies.

Ablation with millisecond pulses has been attempted by placing circular electrodes on pig ventricular myocardium and applying defibrillator shocks of 100-200J [41]. While lesion depths up to 7 mm were achieved, the lesions were not uniformly transmural. Microsecond pulses were successfully used to create nonconducting lesions in pig atrial appendages, although with energies approximately 1,000 times higher than those presented here [40].

The fact that we demonstrate nsPEF ablation here with a fraction of the applied energy suggests that nsPEF ablation is more efficient for ablation than longer pulses. This result is consistent with theoretical analyses that suggest that nsPEF ablation electroporates cells in a tissue more uniformly. It has been shown in modeling studies that the strong fields used for nanosecond pulses electroporate all cells indiscriminately, while the weaker fields associated with longer pulses lead to spatially heterogeneous, incomplete electroporation [26,43]. Recent experiments in rat embryonic cardiomyocytes likewise suggest that nsPEF electroporates cells more uniformly that millisecond pulsed electric fields [44]. While it would be in principle possible to generate pulses that are both strong and long, such pulses would deposit large energies, which would lead to unwanted thermal effects. Nanosecond pulses also have the benefit that it has already been shown that besides ablating cells via necrosis, they can also ablate them via apoptosis [45,46].

### Clinical application of nsPEFs ablation

In this paper, we are demonstrating the principle of nsPEF ablation in isolated hearts. In clinical practice, nsPEF ablation could either be performed via surgical clamps during open heart surgery, or using a catheter (just as RF- or cryoablation). For nsPEF ablation during open heart surgery, surgical clamps similar to those used in RF ablation [47] could be equipped with arrays of penetrating electrodes to allow the creation of extended lesions with a single nsPEF application. The development of this technology is straightforward and we plan to move it towards clinical practice first. For nsPEF catheter ablation, retractable penetrating electrodes would be placed in a catheter similar to those used in RF ablation. The thin, sharpened electrodes can be inserted into the myocardium with small contact force. Note that the wall thickness of the rabbit ventricles (2–4 mm) closely matches that of the human atria, which would be one important target for nsPEF ablation. Ventricular ablation, while performed less commonly, is also a very important target due to the grave risk of ventricular tachyarrhythmias that patients receiving ventricular typically carry. The substantially thicker human ventricles would not pose a special challenge to our nsPEF ablation approach; the electrode length would be increased accordingly. It is reasonable to expect that the consistent cross section of lesions that we observe, and that is due to the translational symmetry of our electrode configuration across the wall, would also be observed in thicker tissue (because the translational symmetry would still be given).

### Arrhythmia induction

An important concern regarding shock application in a clinical setting is that shocks may induce fibrillation in the patient. In our rabbit model, nsPEFs never induced fibrillation that lasted long enough for us to observe it (several seconds would be detectable for us), but it is well-known that larger species such as human, dog, and pig are more prone to the induction of fibrillation [48]. In fact, application of microsecond shocks to pig heart frequently does induce fibrillation. This problem can, however, be addressed by timing the shock application so that it occurs right after the QRS complex (i.e. when all of the myocardium is depolarized) [49].

### Limitations

This study has several limitations. Even though the consistent width of our lesions suggests that recurrence will be low, this has not been tested experimentally. Long-term survival studies that are necessary to address this limitation, and we are currently planning such studies. Also, it would be desirable to have an electrode configuration in which the electrodes don't penetrate the tissue multiple times. We are currently testing an alternative electrode configuration in which one electrode is places on the epicardium and the other electrode on the endocardium. This new configuration is straightforward to implement for open heart surgery, but for a catheter-based approach, there will be challenges related to the alignment of catheters.

### Conclusions

Ablation with nsPEF is a promising alternative to RF and cryoablation that may overcome limitations of current clinical practice. Chronic animal studies and studies in large animal hearts are needed to evaluate the clinical potential of nsPEF ablation.

## References

[1] Khan AR, Khan S, Sheikh MA, Khuder S, Grubb B, Moukarbel GV. Catheter ablation and antiarrhythmic drug therapy as first- or second-line therapy in the management of atrial fibrillation: systematic review and meta-analysis. Circ Arrhythm Electrophysiol. 2014 10;7(5):853–60. 10.1161/CIRCEP.114.001853 25110162

[2] Santangeli P, Di Biase L, Natale A. Ablation versus drugs: what is the best first-line therapy for paroxysmal atrial fibrillation? Antiarrhythmic drugs are outmoded and catheter ablation should be the first-line option for all patients with paroxysmal atrial fibrillation: pro. Circ Arrhythm Electrophysiol. 2014 8;7(4):739–46. 10.1161/CIRCEP.113.000629 25140019

[3] Wu S-H, Jiang W-F, Gu J, Zhao L, Wang Y-L, Liu Y-G, et al Benefits and risks of additional ablation of complex fractionated atrial electrograms for patients with atrial fibrillation: a systematic review and meta-analysis. Int J Cardiol. 2013 10 25;169(1):35–43. 10.1016/j.ijcard.2013.08.083 24083885

[4] Natale A. Advances in catheter-ablation treatment of AF. Nat Rev Cardiol. 2013 2;10(2):63–4. 10.1038/nrcardio.2012.198 23319098

[5] Schleifer JW, Srivathsan K. Ventricular arrhythmias: state of the art. Cardiol Clin. 2013 11;31(4):595–605, ix 10.1016/j.ccl.2013.07.007 24188223

[6] Gal P, Aarntzen AESM, Smit JJJ, Adiyaman A, Misier ARR, Delnoy PPHM, et al Conventional radiofrequency catheter ablation compared to multi-electrode ablation for atrial fibrillation. Int J Cardiol. 2014 10 20;176(3):891–5. 10.1016/j.ijcard.2014.08.034 25156854

[7] Kearney K, Stephenson R, Phan K, Chan WY, Huang MY, Yan TD. A systematic review of surgical ablation versus catheter ablation for atrial fibrillation. Ann Cardiothorac Surg. 2014 1;3(1):15–29. 10.3978/j.issn.2225-319X.2014.01.03 24516794 PMC3904326

[8] Lee G, Sanders P, Kalman JM. Catheter ablation of atrial arrhythmias: state of the art. Lancet Lond Engl. 2012 10 27;380(9852):1509–19. 10.1016/S0140-6736(12)61463-9 23101718

[9] Gerstenfeld EP, Callans DJ, Dixit S, Zado E, Marchlinski FE. Incidence and location of focal atrial fibrillation triggers in patients undergoing repeat pulmonary vein isolation: implications for ablation strategies. J Cardiovasc Electrophysiol. 2003 7;14(7):685–90. 12930245 10.1046/j.1540-8167.2003.03013.x

[10] Yokoyama K, Nakagawa H, Wittkampf FHM, Pitha JV, Lazzara R, Jackman WM. Comparison of electrode cooling between internal and open irrigation in radiofrequency ablation lesion depth and incidence of thrombus and steam pop. Circulation. 2006 1 3;113(1):11–9. 16380552 10.1161/CIRCULATIONAHA.105.540062

[11] Nakagawa H, Yamanashi WS, Pitha JV, Arruda M, Wang X, Ohtomo K, et al Comparison of in vivo tissue temperature profile and lesion geometry for radiofrequency ablation with a saline-irrigated electrode versus temperature control in a canine thigh muscle preparation. Circulation. 1995 4 15;91(8):2264–73. 7697856 10.1161/01.cir.91.8.2264

[12] Bertaglia E, Zoppo F, Tondo C, Colella A, Mantovan R, Senatore G, et al Early complications of pulmonary vein catheter ablation for atrial fibrillation: a multicenter prospective registry on procedural safety. Heart Rhythm. 2007 10;4(10):1265–71. 17905330 10.1016/j.hrthm.2007.06.016

[13] Spragg DD, Dalal D, Cheema A, Scherr D, Chilukuri K, Cheng A, et al Complications of catheter ablation for atrial fibrillation: incidence and predictors. J Cardiovasc Electrophysiol. 2008 6;19(6):627–31. 10.1111/j.1540-8167.2008.01181.x 18462327

[14] Woods CE, Olgin J. Atrial fibrillation therapy now and in the future: drugs, biologicals, and ablation. Circ Res. 2014 4 25;114(9):1532–46. 10.1161/CIRCRESAHA.114.302362 24763469 PMC4169264

[15] Zhao J, Kharche SR, Hansen BJ, Csepe TA, Wang Y, Stiles MK, et al Optimization of Catheter Ablation of Atrial Fibrillation: Insights Gained from Clinically-Derived Computer Models. Int J Mol Sci. 2015;16(5):10834–54. 10.3390/ijms160510834 25984605 PMC4463678

[16] Winkle RA, Mead RH, Engel G, Kong MH, Patrawala RA. Atrial fibrillation ablation using open-irrigated tip radiofrequency: experience with intraprocedural activated clotting times ≤210 seconds. Heart Rhythm. 2014 6;11(6):963–8. 10.1016/j.hrthm.2014.03.013 24681115

[17] Ang R, Domenichini G, Finlay MC, Schilling RJ, Hunter RJ. The Hot and the Cold: Radiofrequency Versus Cryoballoon Ablation for Atrial Fibrillation. Curr Cardiol Rep. 2015 9;17(9):631 10.1007/s11886-015-0631-7 26266757

[18] Khairy P, Chauvet P, Lehmann J, Lambert J, Macle L, Tanguay J-F, et al Lower incidence of thrombus formation with cryoenergy versus radiofrequency catheter ablation. Circulation. 2003 4 22;107(15):2045–50. 12668527 10.1161/01.CIR.0000058706.82623.A1

[19] Jourda F, Providencia R, Marijon E, Bouzeman A, Hireche H, Khoueiry Z, et al Contact-force guided radiofrequency vs. second-generation balloon cryotherapy for pulmonary vein isolation in patients with paroxysmal atrial fibrillation-a prospective evaluation. Europace. 2015 2;17(2):225–31. 10.1093/europace/euu215 25186456

[20] Chen R, Sain NM, Harlow KT, Chen Y-J, Shires PK, Heller R, et al A protective effect after clearance of orthotopic rat hepatocellular carcinoma by nanosecond pulsed electric fields. Eur J Cancer. 1990 2014 10;50(15):2705–13. 10.1016/j.ejca.2014.07.006 25081978

[21] Kotnik T, Miklavcic D. Second-order model of membrane electric field induced by alternating external electric fields. IEEE Trans Biomed Eng. 2000 8;47(8):1074–81. 10943056 10.1109/10.855935

[22] Son RS, Smith KC, Gowrishankar TR, Vernier PT, Weaver JC. Basic features of a cell electroporation model: illustrative behavior for two very different pulses. J Membr Biol. 2014 12;247(12):1209–28. 10.1007/s00232-014-9699-z 25048527 PMC4224743

[23] Saviz M, Faraji-Dana R. Simplified estimation of membrane potentials induced by high-frequency electric signals. Bioimpedance. 2014;69(6):9–13.

[24] Wikswo JP, Lin SF, Abbas RA. Virtual electrodes in cardiac tissue: a common mechanism for anodal and cathodal stimulation. Biophys J. 1995 12;69(6):2195–210. 8599628 10.1016/S0006-3495(95)80115-3 PMC1236459

[25] Gowrishankar TR, Weaver JC. An approach to electrical modeling of single and multiple cells. PNAS. 2003 3 18;100(6):3203–8. 12626744 10.1073/pnas.0636434100 PMC152270

[26] Gowrishankar TR, Weaver JC. Electrical behavior and pore accumulation in a multicellular model for conventional and supra-electroporation. Biochem Biophys Res Commun. 2006 10 20;349(2):643–53. 16959217 10.1016/j.bbrc.2006.08.097 PMC1698465

[27] Mankowski J, Kristiansen M. A review of short pulse generator technology. Plasma Sci IEEE Trans On. 2000;28(1):102–8.

[28] Wang YT, Efimov IR, Cheng Y. Electroporation induced by internal defibrillation shock with and without recovery in intact rabbit hearts. Am J Physiol Heart Circ Physiol. 2012 8 15;303(4):H439–49. 10.1152/ajpheart.01121.2011 22730387 PMC3423145

[29] Kuwahara T, Takahashi A, Okubo K, Takagi K, Yamao K, Nakashima E, et al Oesophageal cooling with ice water does not reduce the incidence of oesophageal lesions complicating catheter ablation of atrial fibrillation: randomized controlled study. Europace. 2014 6;16(6):834–9. 10.1093/europace/eut368 24469436

[30] Pappone C, Oral H, Santinelli V, Vicedomini G, Lang CC, Manguso F, et al Atrio-esophageal fistula as a complication of percutaneous transcatheter ablation of atrial fibrillation. Circulation. 2004 6 8;109(22):2724–6. 15159294 10.1161/01.CIR.0000131866.44650.46

[31] Cummings JE, Schweikert RA, Saliba WI, Burkhardt JD, Kilikaslan F, Saad E, et al Brief communication: atrial-esophageal fistulas after radiofrequency ablation. Ann Intern Med. 2006 4 18;144(8):572–4. 16618954 10.7326/0003-4819-144-8-200604180-00007

[32] Rostamian A, Narayan SM, Thomson L, Fishbein M, Siegel RJ. The incidence, diagnosis, and management of pulmonary vein stenosis as a complication of atrial fibrillation ablation. J Interv Card Electrophysiol. 2014 6;40(1):63–74. 10.1007/s10840-014-9885-z 24626996

[33] Yang H-M, Lai CK, Patel J, Moore J, Chen P-S, Shivkumar K, et al Irreversible intrapulmonary vascular changes after pulmonary vein stenosis complicating catheter ablation for atrial fibrillation. Cardiovasc Pathol. 2007 2;16(1):51–5. 17218215 10.1016/j.carpath.2006.07.007

[34] Holmes DR, Monahan KH, Packer D. Pulmonary vein stenosis complicating ablation for atrial fibrillation: clinical spectrum and interventional considerations. JACC Cardiovasc Interv. 2009 4;2(4):267–76. 10.1016/j.jcin.2008.12.014 19463436

[35] Piccini JP, Daubert JP. Cryoablation of atrial fibrillation. J Interv Card Electrophysiol. 2011 12;32(3):233–42. 10.1007/s10840-011-9603-z 21826506

[36] Arena CB, Szot CS, Garcia PA, Rylander MN, Davalos RV. A Three-Dimensional In Vitro Tumor Platform for Modeling Therapeutic Irreversible Electroporation. Biophys J. 2012 11 7;103(9):2033–42. 10.1016/j.bpj.2012.09.017 23199931 PMC3491727

[37] Davalos RV, Mir LM, Rubinsky B. Tissue Ablation with Irreversible Electroporation. Ann Biomed Eng. 2005 2;33(2):223–31. 15771276 10.1007/s10439-005-8981-8

[38] Jackman WM, Wang XZ, Friday KJ, Roman CA, Moulton KP, Beckman KJ, et al Catheter ablation of accessory atrioventricular pathways (Wolff-Parkinson-White syndrome) by radiofrequency current. N Engl J Med. 1991 6 6;324(23):1605–11. 2030716 10.1056/NEJM199106063242301

[39] Scheinman MM. History of Wolff-Parkinson-White Syndrome. Pacing Clin Electrophysiol. 2005 2 1;28(2):152–6. 15679646 10.1111/j.1540-8159.2005.09461.x

[40] Lavee J, Onik G, Mikus P, Rubinsky B. A novel nonthermal energy source for surgical epicardial atrial ablation: irreversible electroporation. Heart Surg Forum. 2007;10(2):E162–7. 17597044 10.1532/HSF98.20061202

[41] Wittkampf FHM, van Driel VJ, van Wessel H, Neven KGEJ, Gründeman PF, Vink A, et al Myocardial Lesion Depth With Circular Electroporation Ablation. Circ Arrhythm Electrophysiol. 2012 6 1;5(3):581–6. 10.1161/CIRCEP.111.970079 22492429

[42] Neven K, van Driel V, van Wessel H, van Es R, du Pré B, Doevendans PA, et al Safety and feasibility of closed chest epicardial catheter ablation using electroporation. Circ Arrhythm Electrophysiol. 2014 10;7(5):913–9. 10.1161/CIRCEP.114.001607 25156260

[43] Gowrishankar TR, Esser AT, Vasilkoski Z, Smith KC, Weaver JC. Microdosimetry for conventional and supra-electroporation in cells with organelles. Biochem Biophys Res Commun. 2006 3 24;341(4):1266–76. 16469297 10.1016/j.bbrc.2006.01.094

[44] Semenov I, Zemlin C, Pakhomova ON, Xiao S, Pakhomov AG. Diffuse, non-polar electropermeabilization and reduced propidium uptake distinguish the effect of nanosecond electric pulses. Biochim Biophys Acta. 2015 6 22;1848(10 Pt A):2118–25. 10.1016/j.bbamem.2015.06.018 26112464 PMC4554928

[45] Xiao D, Yao C, Liu H, Li C, Cheng J, Guo F, et al Irreversible electroporation and apoptosis in human liver cancer cells induced by nanosecond electric pulses. Bioelectromagnetics. 2013 10;34(7):512–20. 10.1002/bem.21796 23740887

[46] Joshi RP, Schoenbach KH. Bioelectric effects of intense ultrashort pulses. Crit Rev Biomed Eng. 2010;38(3):255–304. 21133836 10.1615/critrevbiomedeng.v38.i3.20

[47] Philpott JM, Zemlin CW, Cox JL, Stirling M, Mack M, Hooker RL, et al The ABLATE Trial: Safety and Efficacy of Cox Maze-IV Using a Bipolar Radiofrequency Ablation System. Ann Thorac Surg. 2015 11;100(5):1541–8. 10.1016/j.athoracsur.2015.07.006 26387721

[48] Panfilov AV. Is heart size a factor in ventricular fibrillation? Or how close are rabbit and human hearts? Heart Rhythm. 2006 7;3(7):862–4. 16818223 10.1016/j.hrthm.2005.12.022

[49] Hargrave B, Downey H, Strange R, Murray L, Cinnamond C, Lundberg C, et al Electroporation-mediated gene transfer directly to the swine heart. Gene Ther. 2013 2;20(2):151–7. 10.1038/gt.2012.15 22456328 PMC3387511

